# Cerebral Ultrasound Time-Harmonic Elastography Reveals Softening of the Human Brain Due to Dehydration

**DOI:** 10.3389/fphys.2020.616984

**Published:** 2021-01-11

**Authors:** Bernhard Kreft, Judith Bergs, Mehrgan Shahryari, Leon Alexander Danyel, Stefan Hetzer, Jürgen Braun, Ingolf Sack, Heiko Tzschätzsch

**Affiliations:** ^1^Institute of Medical Informatics, Charité–Universitätsmedizin Berlin, Berlin, Germany; ^2^Department of Radiology, Charité–Universitätsmedizin Berlin, Berlin, Germany; ^3^Department of Neurology, Charité–Universitätsmedizin Berlin, Berlin, Germany; ^4^Bernstein Center of Advanced Neuroimaging, Charité–Universitätsmedizin Berlin, Berlin, Germany

**Keywords:** brain, elastography, hydration, ultrasound, time-harmonic elastography

## Abstract

Hydration influences blood volume, blood viscosity, and water content in soft tissues – variables that determine the biophysical properties of biological tissues including their stiffness. In the brain, the relationship between hydration and stiffness is largely unknown despite the increasing importance of stiffness as a quantitative imaging marker. In this study, we investigated cerebral stiffness (CS) in 12 healthy volunteers using ultrasound time-harmonic elastography (THE) in different hydration states: (i) during normal hydration, (ii) after overnight fasting, and (iii) within 1 h of drinking 12 ml of water per kg body weight. In addition, we correlated shear wave speed (SWS) with urine osmolality and hematocrit. SWS at normal hydration was 1.64 ± 0.02 m/s and decreased to 1.57 ± 0.04 m/s (*p* < 0.001) after overnight fasting. SWS increased again to 1.63 ± 0.01 m/s within 30 min of water drinking, returning to values measured during normal hydration (*p* = 0.85). Urine osmolality at normal hydration (324 ± 148 mOsm/kg) increased to 784 ± 107 mOsm/kg (*p* < 0.001) after fasting and returned to normal (288 ± 128 mOsm/kg, *p* = 0.83) after water drinking. SWS and urine osmolality correlated linearly (*r* = −0.68, *p* < 0.001), while SWS and hematocrit did not correlate (*p* = 0.31). Our results suggest that mild dehydration in the range of diurnal fluctuations is associated with significant softening of brain tissue, possibly due to reduced cerebral perfusion. To ensure consistency of results, it is important that cerebral elastography with a standardized protocol is performed during normal hydration.

## Introduction

The body water content ranges between 55 and 60% in adults and varies with age, sex, and body constitution, while human brain tissue has a very high water content of about 75% (Mitchell et al., 1945), underlining the importance of adequate cerebral tissue hydration for normal brain function. The human organism can easily adapt to a water deficit of 2–3%; however, beyond this limit, dehydration can impair mental and physical coordination, eventually leading to a fatal breakdown of vital body functions (Ashcroft, 2001). Clinically, three types of dehydration are distinguished: (i) isotonic dehydration, that is loss of body water and salt usually occurring after excessive vomiting, diarrhea or bleeding, (ii) hypotonic dehydration, in which salt loss outweighs water loss, is often associated with the intake of diuretic drugs or kidney damage, and (iii) hypertonic dehydration, often resulting from a lack of water due to fasting or dehydration (Spital, 2007; Bhave and Neilson, 2011; Jablonski, 2012). The diagnosis of chronic dehydration remains challenging since clinical signs such as dry mucous membranes or dry skin are unspecific (Jequier and Constant, 2010) while blood or urine markers can vary markedly among individuals (Armstrong, 2005; Duning et al., 2005; Patterson et al., 2008).

The hydration state of soft tissues affects a variety of biophysical properties, which can be assessed *in vivo* by MRI or ultrasound. While perfusion MRI, flow MRI, and Doppler ultrasound are sensitive to volume and velocity of blood flow, magnetic resonance elastography (MRE) and ultrasound elastography can non-invasively measure stiffness (Sack and Schaeffter, 2018). Hydration-specific imaging markers are potentially important for the management of neurological diseases since dehydration of brain tissue is a frequent and dangerous condition in elderly patients with Alzheimer’s disease or other types of dementia (Lauriola et al., 2018).

Studies using MRI and ultrasound markers show that dehydration reduces cerebral blood flow (Trangmar et al., 2014, 2015), decreases brain parenchymal volume, and increases ventricular volume (Duning et al., 2005; Streitburger et al., 2012). However, to date no studies have been published that investigated the effect of dehydration on cerebral stiffness (CS).

Our hypothesis is that CS changes with dehydration and water drinking similar to stiffness changes reported for other organs (Guo et al., 2018). For instance, it has been shown that liver stiffness increases with water ingestion and hepatic blood flow (Ipek-Ugay et al., 2016; Tzschatzsch et al., 2016b). The opposite effect, i.e., softening, has been observed in the pancreas and spleen (Dittmann et al., 2017) while kidney stiffness has been reported to change only slightly with increasing bladder filling (Gandhi et al., 2020) and hydration (Marticorena Garcia et al., 2018).

In this study, we investigate the effect of dehydration on CS using cerebral time-harmonic elastography (THE), which utilizes multifrequency vibrations induced in the brain by an external driver in combination with transtemporal ultrasound (Tzschatzsch et al., 2018). Cerebral THE has several advantages over MRE such as being available at the bedside and providing instantaneous feedback, which facilitates identification of rapid CS changes. We will exploit this real-time feedback capability of THE to study possible CS changes induced by dehydration of brain tissue after overnight fasting and water drinking. Overall, this study aims at providing insight into the sensitivity of CS to physiological changes in tissue hydration toward a clinical tool for monitoring brain mechanical properties at the bedside.

## Materials and Methods

### Study Design

The study protocol conformed to the guidelines of the Declaration of Helsinki and was approved by the institutional review board of Charité–Universitätsmedizin Berlin (EA1/242/18). All study participants gave their written consent to conduct the experiment as well as for the publication of any potentially identifiable images or data included in this article. Inclusion criteria were absence of any history of cerebral disease or trauma and no impairment of renal function. Insufficient acoustic windows due to high skull thickness cause relatively high dropout rates of around 10% in studies of transcranial ultrasound (Marinoni et al., 1997). To avoid such dropout rates, we checked the acoustic window previously to the measurements. Finally, a total of 12 volunteers (3/9 females/males; mean age of 33 ± 9 years, range: 22–50 years) with a sufficient transcranial bone window were included in the study, while one volunteer did not meet the inclusion criterion. Two volunteers (#5 and #12) were further excluded from statistical group analysis due to incomplete fasting. Volunteer demographic data, including sex, age, and body mass index (BMI) are summarized in Table 1.

**Table 1:** Demographic data, including sex, age, and BMI with group mean and standard deviation (SD) of all volunteers.

Subject #	Sex	Age in years	BMI in kg/m^2^
1	m	25	23.2
2	m	50	20.1
3	f	40	21.5
4	f	27	31.6
5	m	26	20.2
6	m	35	19.9
7	m	46	25.8
8	f	28	20.7
9	m	37	26.2
10	m	22	24.0
11	m	22	24.5
12	m	43	23.6
Mean (SD)		33.4 (9.3)	23.4 (3.5)

Volunteers #5 and #12 were excluded from statistical analysis due to poor compliance with the study protocol.

**Figure 1:**
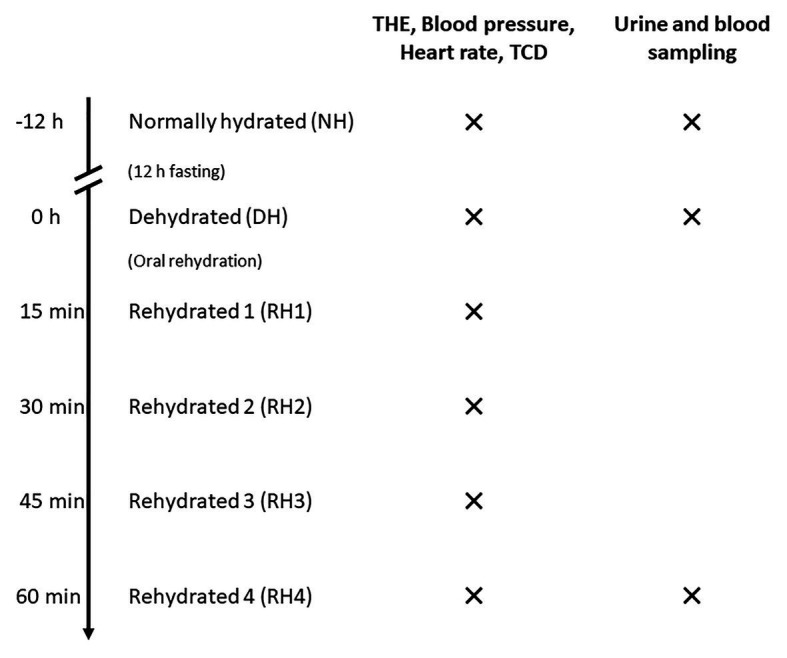
Time sequence of THE examinations in volunteers. Volunteers were first examined in a normally hydrated state (NH). After a 12-h-fasting period, measurements were conducted in the dehydrated state (DH). Then, four sets of data in different hydration states were acquired after oral rehydration (RH1–RH4) at 15-min intervals. For every hydration state, time-harmonic elastography (THE), transcranial Doppler (TCD), and blood pressure and heart rate measurements were performed. Urine and blood for determination of urine osmolality and hematocrit were sampled at hydration states NH, DH, and RH4.

Each participant was investigated on 2 days. On the first day, data were acquired in a normally hydrated (NH) state, defined as drinking 1.5 L water within 5 h prior to the examination. After 12-h overnight fasting, the second set of data (dehydrated, DH) was acquired. Then, the volunteers were asked to drink 12 ml/kg of water within 15 min (Duning et al., 2005). Immediately after drinking water, four sets of data (rehydrated, RH1–RH4) were acquired at 15-min intervals. Thus, a total of six sets of data were acquired for every volunteer. Each dataset consisted of THE, transcranial Doppler (TCD), and recordings of blood pressure and heart rate. Urine and blood were collected at hydration states 1 (NH), 2 (DH), and 6 (RH4) to determine urine osmolality and hematocrit. The time sequence of THE examinations is illustrated in Figure 1.

### Cerebral THE

**Figure 2:**
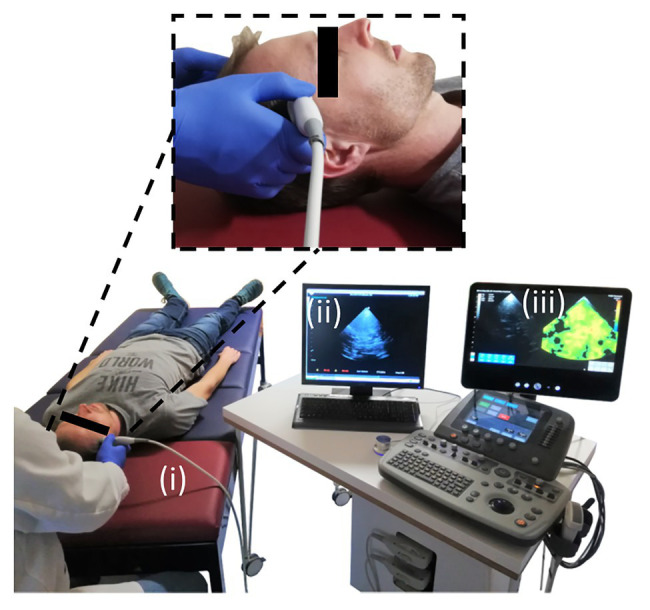
Experimental setup for conduct of cerebral THE. The setup comprised of three main components: (i) the customized patient bed with the vibration plate (red), (ii) the clinical ultrasound scanner, and (iii) the elastography computer with the integrated post-processing software. The magnification shows the positioning of the ultrasound probe over the volunteer’s temporal bone window for transcranial ultrasound.

The setup of cerebral THE including (i) customized vibration bed with vibration plate mounted on a shaker (GAMPT, Merseburg, Germany), (ii) standard clinical ultrasound scanner (SonixMDP, UltraSonix, Scottsdale AZ, United States) equipped with a phased-array transducer (SA4-2/24), and (iii) elastography computer with the integrated post-processing pipeline is illustrated in Figure 2. The volunteers were asked to lie in a supine position with the head on the vibration plate. According to the current guidelines for transcranial ultrasound (Brian D Coley et al., 2012), the probe was positioned for imaging through the temporal bone window, as shown in the magnification in Figure 2. Shear waves were induced in the head by applying a multifrequency waveform comprising six frequencies (27, 33, 39, 44, 50, and 56 Hz). Signal-to-noise-ratio (SNR) in transcranial ultrasound is known to be relatively low compared to abdominal ultrasound. The stability of shear wave speed (SWS) estimation was found to be stable at vibration amplitudes larger than 2 μm. Therefore, we ensured that vibration amplitudes were above this threshold in order to compensate for low SNR. For data acquisition, a basic preset for transcranial Doppler ultrasound was adapted from the UltraSonix-System (Phased Array, Vascular, TCD, 3.3 MHz). To further improve image SNR, number of pulse cycles was increased to two. In addition, the frame rate was adjusted to 80 Hz in order to fulfill the requirements of the post-processing pipeline. For elastography, ultrasound radiofrequency data were acquired over 1 s and transferred to the elastography computer, where the post-processing was performed.

To obtain the tissue displacement caused by multifrequency vibration, the axial phase shift between adjacent frames was calculated. Temporal Fourier transformation was used for the decomposition of the six superimposed frequencies. Thereby, three vibration frequencies were above the Nyquist limit of ½ frame rate = 40 Hz (44, 50, and 56 Hz) and appeared at the aliased spectral positions (36, 30, and 24 Hz, respectively; Tzschatzsch et al., 2016b). Further post-processing was applied for single frequencies. Noise and unwanted motion were suppressed by a spatial 2D Gaussian bandpass filter. The resulting complex valued shear wave field was directional filtered yielding eight single-directional (single wave number) wave images per frequency, which were converted into a single wave speed map by multifrequency phase gradient inversion and weighted averaging over frequency (Tzschatzsch et al., 2016a).

**Figure 3:**
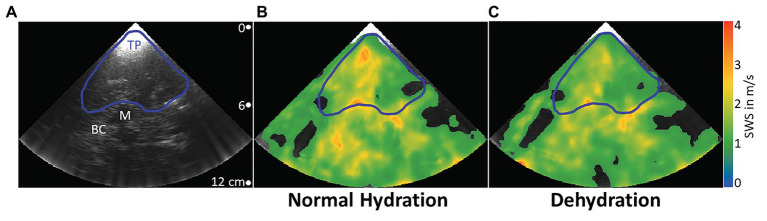
Representative B-mode image and elastograms in normal hydrated and dehydrated states. Transcranial B-mode image **(A)**. Typical landmarks are the temporal parenchyma (blue line, TP), where ROIs were drawn, the mesencephalon (white line, M), which was used as the most prominent landmark, and basal cisterns (BC), a highly vascularized area around the mesencephalon with high echogenicity. Elastograms are shown as color-coded shear wave speed (SWS) maps. In the normally hydrated state **(B)**, mean SWS in the demarcated region of interest is higher than in the dehydrated state **(C)**.

To exclude low SWS-values corresponding to noisy radiofrequency data, we applied a 1 m/s threshold to the elastogram, as explained by Kreft et al. (2020). To determine the average SWS in the temporal lobe parenchyma, a region of interest (ROI) was manually defined based on anatomical landmarks in the B-mode image delimiting that region from the midbrain, such as the butterfly-shaped hypoechogenic mesencephalon and the surrounding hyperechogenic basal cisterns. Areas in the elastogram that corresponded to B-mode artifacts such as reverberation artifacts from the skull were excluded from the ROI. Figure 3 illustrates the drawn ROI in a representative volunteer. For further stabilization, measurements were repeated 10 times. Since motion was negligible, we applied the same ROI to all 10 elastograms for averaging the 10 intra-ROI SWS to one mean SWS-value. It should be noted that this mean SWS value efficiently averaged out the relatively large variability of SWS within a ROI and thus did not reflect intraregional standard deviations. The duration of a continuous examination was no longer than 2 min, which prevented heating of the skull by ultrasound energy absorption.

In order to reduce the expectation bias of the investigator who also analyzed the data, the order of THE acquisitions during all six hydration states was randomly permuted before the ROIs were drawn, and chronological order was restored just after the calculation of SWS.

### Transcranial Doppler Ultrasound

A transcranial Doppler examination was performed with the ultrasound plane aligned for optimal visibility of the middle cerebral artery (MCA). One Doppler spectrum was acquired, which was used to measure mean MCA blood flow at each hydration state over a range of five heartbeats. As TCD is highly dependent on the position and alignment of the MCA in the brain, blood flow measurement in the MCA was not accomplished in all volunteers. TCD was successful in six volunteers (#1, #6, and #8–11), while THE data could be evaluated in all 12 cases.

### Statistical Analysis

All physiological parameters (SWS, cerebral blood flow velocity, blood pressure, heart rate, urine osmolality, and hematocrit) were measured once at three different hydration states (NH, DH, and RH4). Therefore, physiological parameters were considered as independent variables. Correlations between the 30 SWS values (hydration states NH, DH, and RH4 for all 10 volunteers included) and all other parameters were determined by calculating Pearson’s linear correlation coefficients. Correlations between age or body mass index (BMI) and SWS were calculated separately only for NH, DH, and RH4 state. Differences in SWS variation between different hydration states were assessed using Bartlett’s test for equality of variances. Statistical differences between all data measured at different hydration states were tested by one-way ANOVA. Standard deviations for every hydration state were calculated based on the mean values across all subjects. For all *p*-values lower than 0.05, the null hypothesis was rejected.

## Results

A typical B-mode image and corresponding SWS maps obtained through the temporal bone window by cerebral THE at different hydration states in one volunteer are shown in Figure 3. A blue line delimits the selected ROI, while white labels indicate typical anatomical landmarks, including the mesencephalon and basal cisterns.

**Figure 4:**
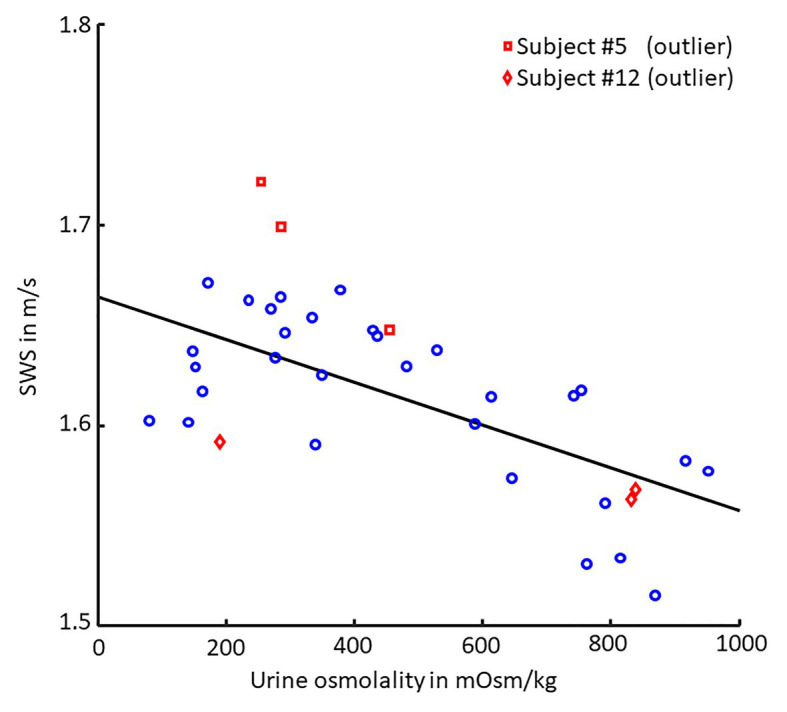
Scatter plot of SWS vs. urine osmolality. Pearson’s linear correlation between shear wave speed (SWS) and urine osmolality (30 values each, *p* < 0.001, *r* = −0.68). Linear regression is shown as black line. Volunteers #5 (□, red) and #12 (◊, red) were excluded from correlation analysis as they failed to comply with the fasting protocol.

**Figure 5:**
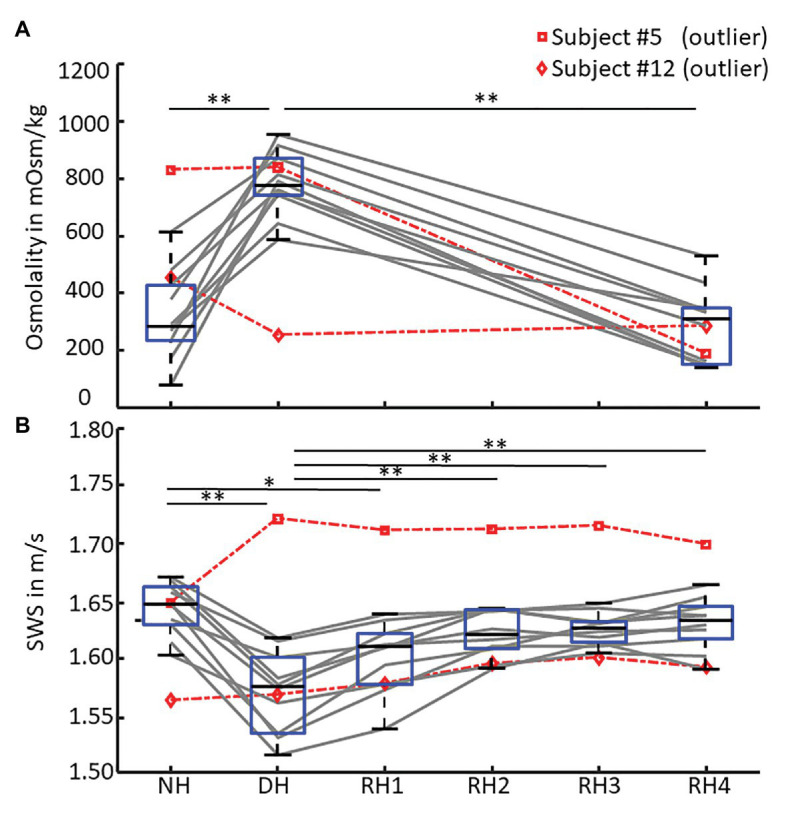
SWS and urine osmolality changes during de‐ and rehydration. Boxplots of **(A)** urine osmolality for three hydration states – normal hydration (NH), dehydration (DH), and rehydration (RH4) and **(B)** mean shear wave speed (SWS) across all hydration states measured in each volunteer. Urine osmolality increased after dehydration and decreased to normal hydration values after 1 h of rehydration. Conversely, SWS significantly decreased after 12 h of fasting and returned to normal hydration values within 30 min of oral rehydration. Volunteers #5 (□, dashed red) and #12 (◊, dashed red) were excluded from statistical group analysis due to incomplete fasting, as reflected in urine osmolality and confirmed by them after the experiment. Significant differences between the groups are indicated by ^*^p < 0.05 and ^**^p < 0.001.

We found a moderate linear correlation between SWS and urine osmolality (*r* = −0.68, *p* < 0.001), as shown in the scatter plot in Figure 4, while SWS did not correlate with any other physiological parameter. Blood pressure, heart rate, cerebral blood flow velocity, and hematocrit values did not change with hydration. In contrast, urine osmolality increased from a normal hydration value of 324 ± 148 mOsm/kg to 784 ± 107 mOsm/kg in the dehydrated state (*p* < 0.001), as illustrated in Figure 5A. In two subjects (#5 and #12), changes in urine osmolality deviated from those observed in the other volunteers, which was attributed to incomplete fasting. As the two volunteers later confirmed this, they were excluded from the statistical group analysis. After water drinking, urine osmolality decreased to 288 ± 128 mOsm/kg (*p* < 0.001), which corresponded to normal hydration values (*p* = 0.83). Inversely to urine osmolality, SWS was found to decrease with dehydration from 1.64 ± 0.02 m/s to 1.57 ± 0.04 m/s (*p* < 0.001; Figure 5B). Within 30 min of water ingestion, SWS increased again to 1.62 ± 0.02 m/s (*p* < 0.001) and remained unchanged for another 30 min, where SWS reached a plateau. The plateau value was similar to normal hydration values (all *p* > 0.9). The variability in SWS was not different between hydration states (*p* = 0.14). All data are summarized in Table 2.

**Table 2:** Group mean values (standard deviation) across volunteers (#5 and #12 excluded) for all physical parameters obtained including shear wave speed (SWS), blood pressure, heart rate, cerebral blood flow velocity, and urine osmolality.

Parameters	NH	DH	RH1	RH2	RH3	RH4
SWS in m/s	1.64 (0.02)	1.57 (0.04)	1.60 (0.03)	1.62 (0.02)	1.63 (0.01)	1.63 (0.02)
Blood pressure in mmHg	131/79 (10/6)	127/75 (7/6)	123/73 (7/9)	123/74 (7/8)	125/77 (6/8)	125/76 (9/9)
Heart rate in bpm	68 (10)	67 (9)	60 (10)	63 (11)	62 (12)	60 (10)
Cerebral blood flow velocity in cm/s	68.3 (7.7)	71.7 (4.4)	68.3 (4.8)	69.4 (6.6)	65.2 (4.6)	68.5 (4.1)
Urine osmolality in mOsm/kg	324 (148)	784 (107)	-	-	-	288 (128)
Hematocrit in %	42.4 (3)	42.2 (2.8)	-	-	-	42.7 (3.3)

All parameters were measured for normal hydration (NH), dehydration (DH), and four times during rehydration (RH1–RH4).

## Discussion

This study shows for the first time that de‐ and rehydration of brain tissue influence brain stiffness *in vivo*.

In healthy subjects, urine osmolality increases with hypertonic dehydration and decreases with oral rehydration. Conversely, in patients with renal dysfunction or chronic kidney disease, when the kidneys’ ability to concentrate urine is impaired, this parameter is less markedly affected by dehydration (Roscoe, 1964; Tabibzadeh et al., 2019). In our study, two volunteers were identified as outliers not complying with the fasting protocol due to their urine osmolality. Notably, changes in SWS were still in line with the changes in urine osmolality observed in these two volunteers, providing further evidence for the high sensitivity of SWS to urine osmolality.

Unlike SWS and urine osmolality, other physiological markers such as blood pressure, heart rate, and hematocrit as well as cerebral blood flow velocity were not observed to be affected by dehydration. Other studies found a slight decrease in blood pressure and cerebral blood flow (Trangmar et al., 2014, 2015; Tsai et al., 2018; Watso and Farquhar, 2019) and an increase in heart rate variability (Castro-Sepulveda et al., 2014) upon mild hypertonic dehydration. However, these studies also measured vessel diameter, which is a critical parameter in assessing cerebral blood flow and could not be reliably assessed in this study. Additionally, the authors of these studies focused on dehydration during physical activity while our measurements were performed under resting conditions, which are characterized by relatively stable physiological parameters.

It is well-known that dehydration decreases cerebral blood volume and blood flow (Duning et al., 2005; Streitburger et al., 2012; Ryding, 2017; Sonig et al., 2020). Blood volume and blood flow in turn influence stiffness. Different studies have addressed the relationship between cerebral blood flow and CS (Guo et al., 2018). For example, studying regional variation of cerebral perfusion in deep gray matter using MRE, we found a direct and positive correlation between perfusion pressure and CS (Tzschatzsch et al., 2016b; Hetzer et al., 2018). Similarly, experimentally induced hypercapnia was associated with a synchronous increase in cerebral blood flow and CS, as revealed by MRE (Hetzer et al., 2019) and THE (Kreft et al., 2020). Previous work also revealed that the Valsalva maneuver causes an increase in CS on a short time scale in the order of seconds (Tzschatzsch et al., 2018). Taken together, this previous evidence suggests that lower cerebral blood flow, as a result of dehydration, reduces CS, which is consistent with the observations made in our study. However, with 4.4 ± 1.7%, the observed effect of dehydration is relatively small, which may be attributable to two factors: first, tissue stiffness is only indirectly linked to blood perfusion through poroelastic interactions such as have been described for *in vivo* brain tissue (McGarry et al., 2015, 2019; Parker, 2017; Lilaj et al., 2020). Second, autoregulation of cerebral blood volume, perfusion pressure, and intracranial pressure only occurs across a small range of biophysical property changes and possibly compensates for minor changes in CS (Donnelly et al., 2015; Moerman and De Hert, 2019). Nevertheless, the observed decrease in SWS from normal hydration to dehydration is significant and potentially adds to the variability of CS measured in patients. Therefore, we recommend that cerebral elastography be performed under normal hydration conditions.

Our study has limitations. Since we did not record any parameters to determine renal function, the urine osmolality we measured, although correlated with SWS, can only be considered as indirect measurements of body tissue dehydration. In TCD measurements, the exact transducer positioning relative to flow direction influences the measured blood velocity value. Therefore, variability of TCD is relatively high and operator dependent. Our experimental setup was optimized for THE leading to a relatively high drop-out rate of 6 from 12 and limited statistical power of our TCD data for the comparison to published values of cerebral blood flow during de‐ and rehydration (Trangmar et al., 2014, 2015). Additionally, our volunteers did not observe a standardized diet *before* fasting, which may have led to variability in our SWS data due to individual adaptation mechanisms. Further biological confounders of brain stiffness should be investigated by cerebral THE in a larger group of volunteers observing standardized conditions.

In summary, CS was measured by cerebral THE in a group of healthy volunteers who fasted overnight to induce mild hypertonic dehydration of brain tissue. We found dehydration to cause a slight decrease in CS on the order of 4.4%. CS increased to normal values within 1/2 h of drinking water. CS correlated with urine osmolality but not with hematocrit. Brain softening due to dehydration might be explained by reduced cerebral perfusion in agreement with prior findings of MRE and THE on the correlation between cerebral perfusion and CS. To minimize variability of CS values in a standardized protocol of cerebral elastography, examiners should make sure that volunteers or patients are in normal hydration states during the examination.

## Data Availability Statement

All data needed to evaluate the conclusions drawn in the article have been presented in the manuscript. Additional data may be requested from the authors.

## Ethics Statement

The studies involving human participants were reviewed and approved by Institutional Review Board of Charité ‐ Universitätsmedizin Berlin. The patients/participants provided their written informed consent to participate in this study.

## Author Contributions

All authors contributed to the conception and design of the study and formed the hypothesis. BK, HT, MS, and SH acquired the data. BK, HT, and LD analyzed the data. BK, HT, LD, and IS drafted the manuscript. All authors carried out critical revision of the manuscript equally.

### Conflict of Interest

The authors declare that the research was conducted in the absence of any commercial or financial relationships that could be construed as a potential conflict of interest.
